# AI-Enabled Physical Activity Intervention Strategies for Children and Adolescents: A Scoping Review

**DOI:** 10.3390/children13081114

**Published:** 2026-08-20

**Authors:** Junling Zhao, Jiandong Huang, Zhaoyang Shang, Zhongkai He

**Affiliations:** 1 School of Public Health, Peking University, Beijing 100191, China; junling@pku.edu.cn; 2 China Center for Health Development Studies, Peking University, Beijing 100191, China; 3 School of Control and Computer Engineering, North China Electric Power University, Beijing 102206, China; 120241280105@ncepu.edu.cn; 4 Department of Applied Mathematics and Informatics, Shenzhen MSU-BIT University, Shenzhen 518172, China; 2120250031@smbu.edu.cn; 5 Department of Physical Education, Peking University, Beijing 100871, China

**Keywords:** artificial intelligence, machine learning, physical activity, sedentary behaviour, children, adolescents, scoping review

## Abstract

**Highlights:**

**What are the main findings?**

- Seven studies published between 2022 and 2026 applied diverse artificial intelligence techniques—most often computer vision, followed by generative AI and large language models—through immersive, conversational, game-based, and robotic formats.
- The evidence comprised three controlled studies and four single-arm pilot or feasibility studies; only one was a full-scale randomized controlled trial.

**What are the implications of the main findings?**

- AI technologies offer potential mechanisms for personalized content and adaptive feedback, but current evidence is insufficient to determine whether these mechanisms improve engagement, physical activity, or health outcomes beyond comparable non-AI interventions.
- Future work should use adequately powered controlled designs, objective and standardized measures, AI-specific reporting, and explicit safeguards for privacy, fairness, and safety.

**Abstract:**

Background/Objectives: Most children and adolescents do not meet recommended physical activity (PA) levels. Artificial intelligence (AI) may enable personalized and adaptive interventions, but evidence across delivery formats remains unclear. This scoping review mapped AI-enabled PA interventions for children and adolescents aged 6–18 years. Methods: Following PRISMA-ScR and Joanna Briggs Institute guidance, six databases were searched on 24 May 2026 for English-language studies published since 2018. Eligible studies used AI in intervention delivery and empirically evaluated at least one participant-level PA-related outcome following exposure to the intervention. The review protocol was registered during data analysis. Results: Seven studies published between 2022 and 2026 were included. Primary AI techniques were computer vision (n = 3), generative AI or large language models (n = 2), reinforcement learning (n = 1), and evolutionary computation (n = 1). Interventions included immersive systems (n = 2), conversational agents (n = 2), exergames or serious games (n = 2), and a robot (n = 1). Three studies used controlled designs and four were single-arm pilot or feasibility studies. Conclusions: AI supported movement recognition, content generation, personalization, and adaptive feedback. However, the small, heterogeneous, and predominantly early-stage evidence base precludes conclusions about effectiveness. Rigorous controlled studies and transparent AI-specific reporting are needed.

## 1. Introduction

Regular physical activity (PA) is essential for the healthy physical, cognitive, and psychosocial development of children and adolescents, and its benefits extend into adult life [1,2]. The World Health Organization (WHO) recommends that children and adolescents aged 5–17 years engage in an average of at least 60 min of moderate-to-vigorous physical activity (MVPA) per day [3]. Despite this recommendation, most young people worldwide do not reach the recommended level; a pooled analysis of population-based surveys estimated that approximately 81% of adolescents aged 11–17 years were insufficiently active [4]. Insufficient PA during childhood and adolescence is associated with a higher risk of obesity and being overweight, unfavourable cardiometabolic profiles, and reduced psychosocial well-being, and these risks frequently persist into adulthood [5,6]. High levels of sedentary behaviour, including prolonged screen time, further increase these risks [7]. Promoting PA and reducing sedentary behaviour in this population therefore remain priorities for public health [3,8].

A wide range of interventions has been developed to increase PA among children and adolescents, including school-based programmes, physical education curricula, and behavioural counselling [9,10]. Although such interventions can produce measurable benefits, their effects are often modest, inconsistent, and difficult to sustain over time [10,11]. Frequently reported limitations include limited personalization to the individual user, insufficient engagement and intrinsic motivation, restricted scalability, and dependence on supervision by trained staff [11,12]. These limitations have prompted growing interest in digital and technology-based approaches—such as wearable activity trackers, mobile applications, active video games (exergames), and immersive virtual and augmented reality—that aim to make PA more engaging, accessible, and individually tailored [12,13,14,15].

Artificial intelligence (AI) has recently emerged as a means of further enhancing the personalization and adaptability of these approaches [16,17]. AI refers to computational methods—including machine learning, deep learning, computer vision, natural language processing, and reinforcement learning—that enable systems to learn from data, recognize patterns, and adapt their outputs without relying solely on predefined rules [16]. In pediatric health, AI has been applied to tasks ranging from diagnosis to risk prediction, and its use is expanding rapidly [17,18]. Within the PA domain, AI provides functions that fixed, predefined programmes cannot offer: it can analyze movement through pose estimation and motion recognition, generate individually adapted exercise content, deliver conversational coaching and feedback, and adjust task difficulty in real time according to a user’s performance [12,15]. These capabilities may help to address the engagement, personalization, and scalability gaps that constrain conventional PA interventions.

Previous reviews have examined individual technology-based strategies for promoting PA in young people, including wearable activity trackers [13], exergames [14], gamification [19], and immersive virtual reality [15]. A preliminary search of PubMed, Web of Science, and the reference lists of relevant reviews identified these modality-specific syntheses but no review that mapped AI as an enabling technology across multiple PA-intervention formats. AI components, when present in earlier reviews, were generally described incidentally rather than analyzed as the central concept. Relevant primary studies are dispersed across the health-sciences and computer-science studies and differ substantially in design, terminology, and delivery format. This dispersion and heterogeneity make it difficult to determine how AI functions within interventions and where empirical evidence is concentrated or absent.

A scoping review is well suited to this situation because it maps an emerging and heterogeneous body of evidence, clarifies concepts, and identifies research gaps rather than estimating the effectiveness of a narrowly defined intervention [20,21,22]. Accordingly, this review systematically identified and characterized AI-enabled PA intervention strategies for children and adolescents aged 6–18 years. Guided by the Population-Concept-Context (PCC) framework, it addressed four questions: (1) Which AI techniques have been used? (2) What functional role does AI perform within each intervention? (3) Which populations, settings, intervention formats, and study designs have been examined? (4) Which PA-related behavioural, experiential, fitness, and health outcomes have been measured? The resulting map is intended to inform the design and evaluation of future AI-enabled PA interventions for young people.

## 2. Materials and Methods

This scoping review was designed and reported in accordance with the Preferred Reporting Items for Systematic Reviews and Meta-Analyses extension for Scoping Reviews (PRISMA-ScR) [23]. The methodological process followed the framework originally proposed by Arksey and O’Malley [20], subsequently refined by Levac et al. [24], and the updated guidance issued by the Joanna Briggs Institute (JBI) [22]. A scoping review approach was selected because the evidence on artificial intelligence (AI)-enabled physical activity (PA) interventions for children and adolescents is emerging, methodologically heterogeneous, and technologically diverse; the aim was therefore to map the nature, range, and characteristics of the available evidence and to identify knowledge gaps, rather than to estimate the effectiveness of a specific intervention, a purpose for which a scoping review is the appropriate method [21].

### 2.1. Protocol and Registration

A review protocol describing the research question, eligibility criteria, search strategy, and data-charting procedures was registered with the International Platform of Registered Systematic Review and Meta-Analysis Protocols (INPLASY) on 19 June 2026 (registration number INPLASY202660095; https://doi.org/10.37766/inplasy2026.6.0095). Registration occurred after the database search and screening, during data analysis. Following registration, a post hoc methodological amendment required included studies to report at least one participant-level PA-related outcome following exposure to the intervention; the study-selection counts and synthesis were updated accordingly.

### 2.2. Eligibility Criteria

Eligibility criteria were structured using the Population-Concept-Context (PCC) framework [22]. The population was children and adolescents aged 6–18 years; mixed-age samples were eligible only when data for participants within this range were separately extractable. The concept was an AI-enabled intervention intended to promote PA or reduce sedentary behaviour. The context was unrestricted and included home, school, clinical, laboratory, and community settings.

For this review, an intervention was operationally defined as AI-enabled when an AI method was a substantive component that generated or selected intervention content, personalized recommendations, classified or monitored movement for feedback, adapted task difficulty or coaching, or otherwise informed intervention delivery or decision-making. Offline AI analysis alone was insufficient unless its output was incorporated into an intervention delivered to participants and the study evaluated a participant-level PA-related outcome following that exposure. Conventional fixed-rule algorithms alone were not considered AI; hybrid systems were eligible when their AI component met the operational definition, and human-in-the-loop systems were eligible with the human role charted explicitly. Sources were included if they (a) enrolled children or adolescents aged 6–18 years, with mixed-age samples eligible only when data for participants within this range were separately extractable; (b) met the operational AI definition; (c) empirically evaluated at least one participant-level PA-related outcome following exposure to the AI-enabled intervention, whether primary or secondary; (d) were peer-reviewed full journal articles or full-length conference papers with accessible full text and empirical results; (e) were published in English, a restriction applied because the review team lacked resources for reliable translation and duplicate screening in other languages; and (f) were published from 1 January 2018 onward, a limit selected to capture contemporary AI methods used in health interventions. PA-related outcomes comprised PA volume or intensity, sedentary behaviour or screen time, energy expenditure or physical fitness, anthropometric outcomes linked to a PA intervention, PA-specific participation, enjoyment or willingness, and feasibility or acceptability of PA delivery.

Sources were excluded if they were reviews, protocols without results, single-subject case reports, abstracts, posters, extended abstracts without a full empirical report, or non-peer-reviewed trial-registry, preprint, or grey-literature records; targeted an ineligible age group; lacked an eligible AI role; evaluated only a prototype or modelling method without participant exposure to the AI-enabled intervention; did not report a participant-level PA-related outcome following intervention exposure; or reported only rehabilitation, functional recovery, cognition, emotion, or nutrition without an eligible PA-related outcome. Rehabilitation studies were excluded when the primary purpose was recovery of a specific impairment rather than PA promotion or sedentary-behaviour reduction. Six standardized full-text exclusion codes were used: E1, population not eligible; E2, no eligible AI component; E3, no eligible PA-related outcome; E4, ineligible publication type; E5, non-English language; and E6, outside the publication window. When more than one reason applied, one code was assigned using the hierarchy E1, E2, E3, E4, E5, and E6.

### 2.3. Information Sources

Six electronic bibliographic databases were searched: PubMed, Web of Science Core Collection, Scopus, Embase, IEEE Xplore, and the ACM Digital Library. IEEE Xplore and the ACM Digital Library were included to capture eligible full conference papers indexed in engineering and computing databases. The final search was executed on 24 May 2026. Study identification was limited to records indexed in these six databases; trial registries, preprint servers, and other grey-literature sources were not searched.

### 2.4. Search Strategy

The executed search combined three concept blocks using Boolean operators: (i) AI and related computational methods; (ii) PA, exercise, and sedentary behaviour; and (iii) children and adolescents. Terms within each block were combined with OR, and the blocks were combined with AND. The review team developed the strategy and adapted the terms, phrase handling, fields, operators, and limits to each database. Date (from 1 January 2018) and language (English) limits were applied. Table 1 reproduces the full PubMed strategy as executed rather than a simplified conceptual summary. Complete database-specific strategies, search dates, limits, export formats, and record counts are reported in Supplementary Tables S1–S6. Conference proceedings were retrieved through IEEE Xplore and the ACM Digital Library and through conference records indexed in the other databases.

### 2.5. Study Selection Process

**Figure 1:**
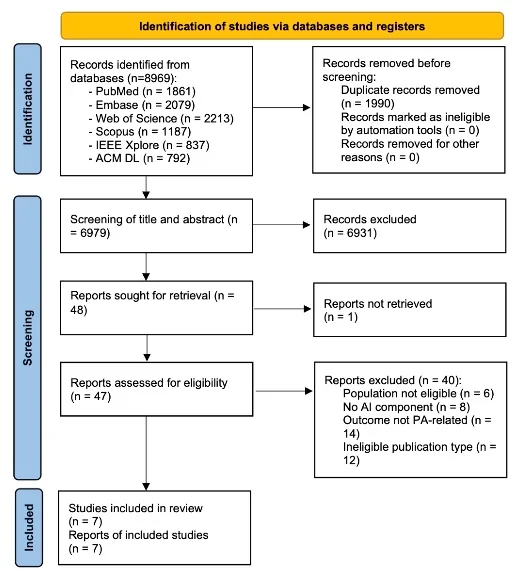
PRISMA-ScR flow diagram of the study selection process (database searches only). PA, physical activity; AI, artificial intelligence; ACM DL, ACM Digital Library.

All records were exported to reference-management software, and duplicates were removed. Screening was performed in two stages using Rayyan (Rayyan Systems Inc., Cambridge, MA, USA) [25]. Two reviewers independently screened titles and abstracts and then assessed retained full texts against the eligibility criteria while blinded to one another’s decisions. Disagreements were resolved through discussion and consensus, with a third reviewer consulted when required. Reports that could not be obtained were recorded as not retrieved. For each excluded full text, reviewers assigned one exclusion code using the prespecified hierarchy reported in Section 2.2. The selection process is summarized in Figure 1.

### 2.6. Data Charting Process

The research team charted the data using a standardized form in Microsoft Excel for Mac, version 16.101.3 (Microsoft Corporation, Redmond, WA, USA). Core bibliographic, participant, intervention, and outcome fields were specified before final charting; the form was pilot-tested and refined iteratively to capture the functional and technical characteristics of AI consistently. One reviewer charted the data and a second reviewer independently verified all entries. Discrepancies were resolved through discussion, and unavailable variables were recorded as not reported.

### 2.7. Data Items

The core variables were author, year, country and setting, study design and comparator, participant characteristics, intervention dose, AI technique, intervention format, PA-related outcomes, measurement tools, and principal findings. AI-specific variables were also charted where reported: model name and version, architecture, training and validation data, pretrained or study-specific status, input data or sensors, AI output or decision, functional role, personalization mechanism, timing of adaptation, model performance, AI-specific comparator, human oversight, error handling and safety constraints, data storage and privacy safeguards, bias or fairness assessment, and explainability or transparency. Participant exposure to AI was classified as direct interaction or AI-generated content delivered through a human. Hybrid systems were assigned one primary technique and format for mapping, with all additional components described narratively. The expanded form and study-level AI-function table are provided in Supplementary Tables S8 and S9.

### 2.8. Critical Appraisal of Individual Sources of Evidence

A formal critical appraisal of methodological quality or risk of bias was not undertaken because the objective was to map the breadth and characteristics of the evidence rather than synthesize effect estimates [22,23]. Study designs are therefore reported descriptively and are not treated as evidence-quality tiers. Outcome findings are presented as results reported by the source studies and are not used to infer comparative effectiveness across the evidence base.

### 2.9. Synthesis of Results

Charted data were synthesized descriptively and narratively and presented in tables and evidence maps. Studies were grouped by primary AI technique, primary intervention format, target population, PA-related outcome category, and study design. Outcome categories were non-mutually exclusive. The technique–format map assigned each hybrid system to the component most central to intervention delivery, while Supplementary Table S9 retained the complete functional description. In the evidence-gap map, colour distinguishes study-design categories only and does not indicate methodological quality or risk of bias.

## 3. Results

### 3.1. Selection of Sources of Evidence

The searches identified 8969 records (PubMed, n = 1861; Embase, n = 2079; Web of Science Core Collection, n = 2213; Scopus, n = 1187; IEEE Xplore, n = 837; ACM Digital Library, n = 792). After removal of 1990 duplicates, 6979 records underwent title and abstract screening, of which 6931 were excluded. Forty-eight reports were sought for full-text retrieval; one was not retrieved, and 47 were assessed for eligibility. Forty reports were excluded: ineligible population (E1, n = 6), no eligible AI component (E2, n = 8), no eligible PA-related outcome (E3, n = 14), and ineligible publication type (E4, n = 12). Seven studies met all criteria and were included (Figure 1).

### 3.2. Characteristics of the Included Studies

**Figure 2:**
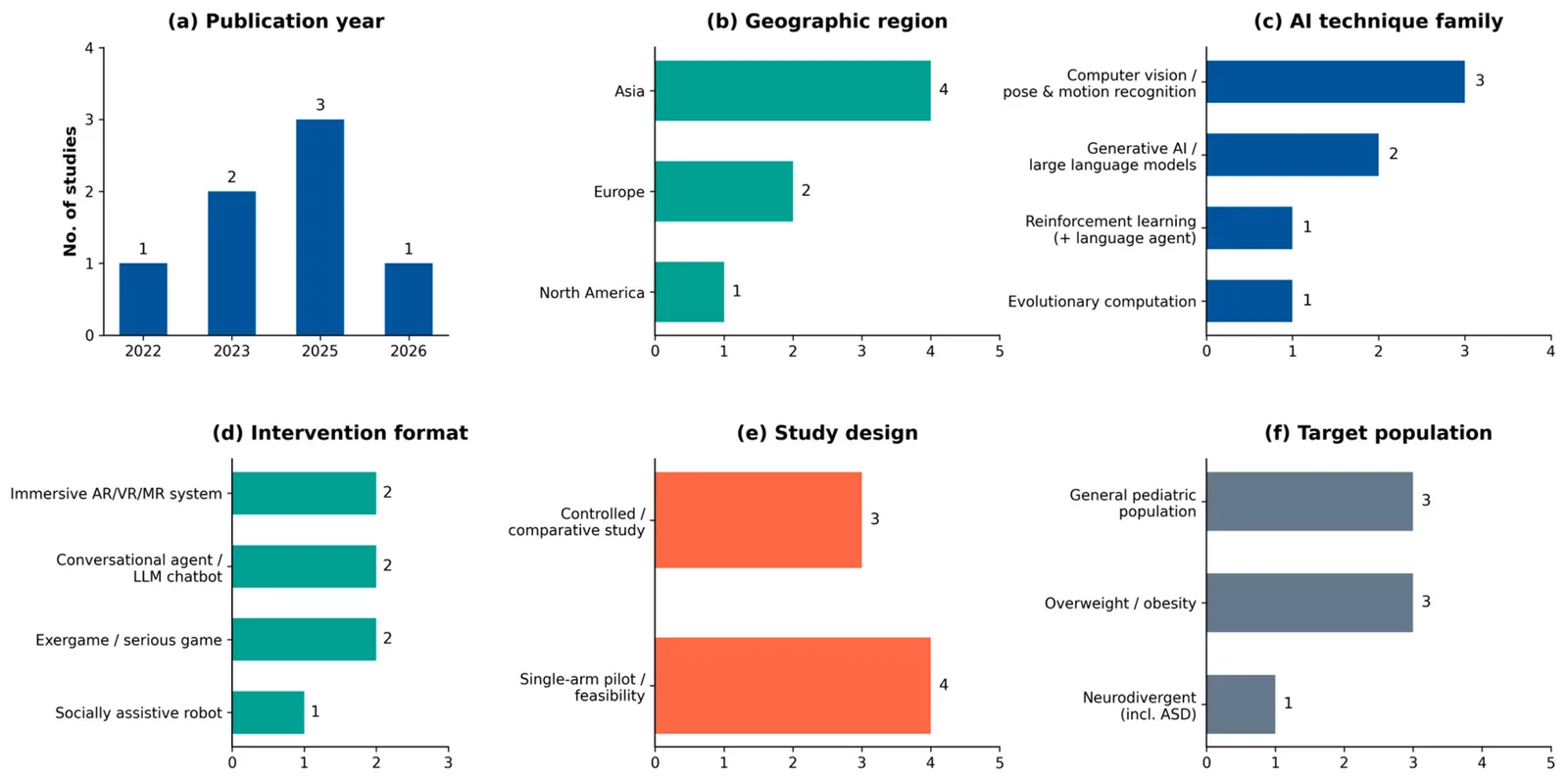
Characteristics of the seven included studies: (**a**) publication year; (**b**) geographic region; (**c**) primary AI technique family; (**d**) primary intervention format; (**e**) study design; and (**f**) target population. Each study was assigned one primary category in panels (**c**–**f**), although hybrid components are described in Supplementary Table S9. Turkey is grouped under Asia (Western Asia).

The seven included studies were published between 2022 and 2026: one in 2022, two in 2023, three in 2025, and one in 2026 (Figure 2a) [26,27,28,29,30,31,32]. Four were conducted in Asia [26,28,29,31], two in Europe [27,30], and one in North America [32] (Figure 2b). Five reports were journal articles [26,28,29,31,32] and two were full conference papers [27,30]. Participant samples ranged from 10 to 227. All seven sources used participant data to evaluate a PA-related behavioural, fitness, experiential, or feasibility outcome following exposure to the intervention. Detailed characteristics are presented in Table 2.

### 3.3. AI Approaches and Techniques

Four primary AI technique families were represented (Figure 2c). Computer vision or pose and motion recognition was most common (three studies) and supported camera-based gesture recognition, action-controlled gameplay, and real-time exercise assessment [26,28,30]. Generative AI or large language models were used in two studies to create parent-mediated activity plans or context-aware student feedback [29,32]. Deep reinforcement learning trained an adaptive transformer-based virtual coaching agent in one study [31], and evolutionary computation was used in one study to select individualized game missions and messages [27]. These roles differed in whether AI operated periodically or in real time and whether participants interacted directly with the AI or received its output through a parent; study-level details are reported in Supplementary Table S9.

### 3.4. Intervention Formats and Delivery

The primary intervention formats were immersive AR/VR systems (two studies) [28,31], conversational agents or chatbots (two studies) [29,32], and exergames or serious games (two studies) [26,27], followed by a socially assistive robot [30] (Figure 2d). Delivery occurred across home, school, laboratory, and supervised exercise contexts. Several interventions combined AI with non-AI components, including activity trackers [27], predefined exercise demonstrations [30], parent delivery [29], rule-based goal attainment [32], or professional-coach templates [31].

### 3.5. Target Populations

Three studies addressed general pediatric or school populations [28,30,32], three focused on children or adolescents who were overweight or obese [26,27,31], and one enrolled children with autism spectrum disorder [29] (Figure 2f). Reported ages ranged from 6 to 17 years.

### 3.6. PA-Related Outcomes and Measurement

PA-related outcomes were heterogeneous and categories were not mutually exclusive. Three studies evaluated PA volume through tracker-derived steps [27], a validated questionnaire [29], or self-report [32]. Energy expenditure or physical fitness was evaluated in two studies [26,31], and sedentary behaviour or screen time in two [27,32]. Participation, enjoyment, or willingness was reported in three studies [28,30,31], and feasibility or acceptability in three [27,29,32]. Individual controlled studies reported differences in calorie expenditure and fitness [26], questionnaire-measured PA [29], or body composition and fitness [31]; whereas, other studies primarily described feasibility, enjoyment, or adaptive delivery. Because designs, comparators, measures, and intervention durations differed and no formal critical appraisal was performed, these source-level findings were not interpreted as pooled or conclusive evidence of effectiveness.

### 3.7. Study Designs

Three studies used controlled designs: a five-arm randomized controlled trial [31], a randomized two-group feasibility study [29], and a randomized comparative trial [26]. Four were single-arm pilot or feasibility evaluations [27,28,30,32] (Figure 2e). Only the five-arm VR sports study was a large trial with follow-up [31]; the remaining studies were generally small, short, or focused on feasibility and technical function. These categories describe study design only and do not imply methodological quality or risk of bias.

### 3.8. Mapping of the Evidence and Evidence Gaps

#### 3.8.1. AI Technique by Intervention Format

**Figure 3:**
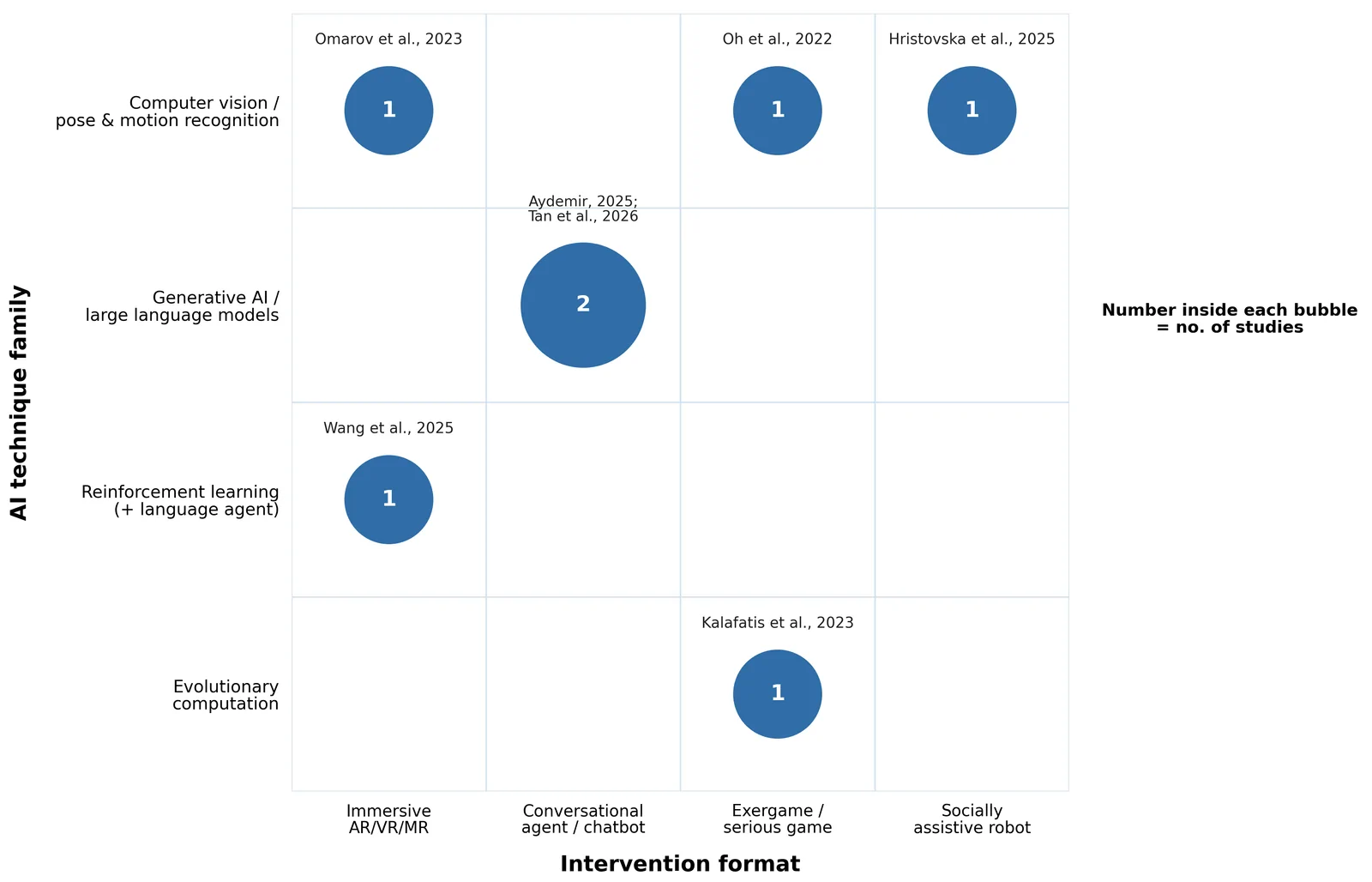
Cross-tabulation of primary AI technique family by primary intervention format (n = 7).

The included studies were cross-tabulated by primary AI technique and primary intervention format (Table 3; Figure 3). Computer vision appeared in immersive AR [28], an exergame [26], and a socially assistive robot [30]. Generative AI or large language models were used in conversational formats [29,32]; reinforcement learning was used in immersive VR [31]; and evolutionary computation supported a serious game [27]. Five of the six represented technique–format cells contained one study, and the conversational generative-AI cell contained two, leaving most possible combinations unexamined.

#### 3.8.2. Evidence-Gap Map

**Figure 4:**
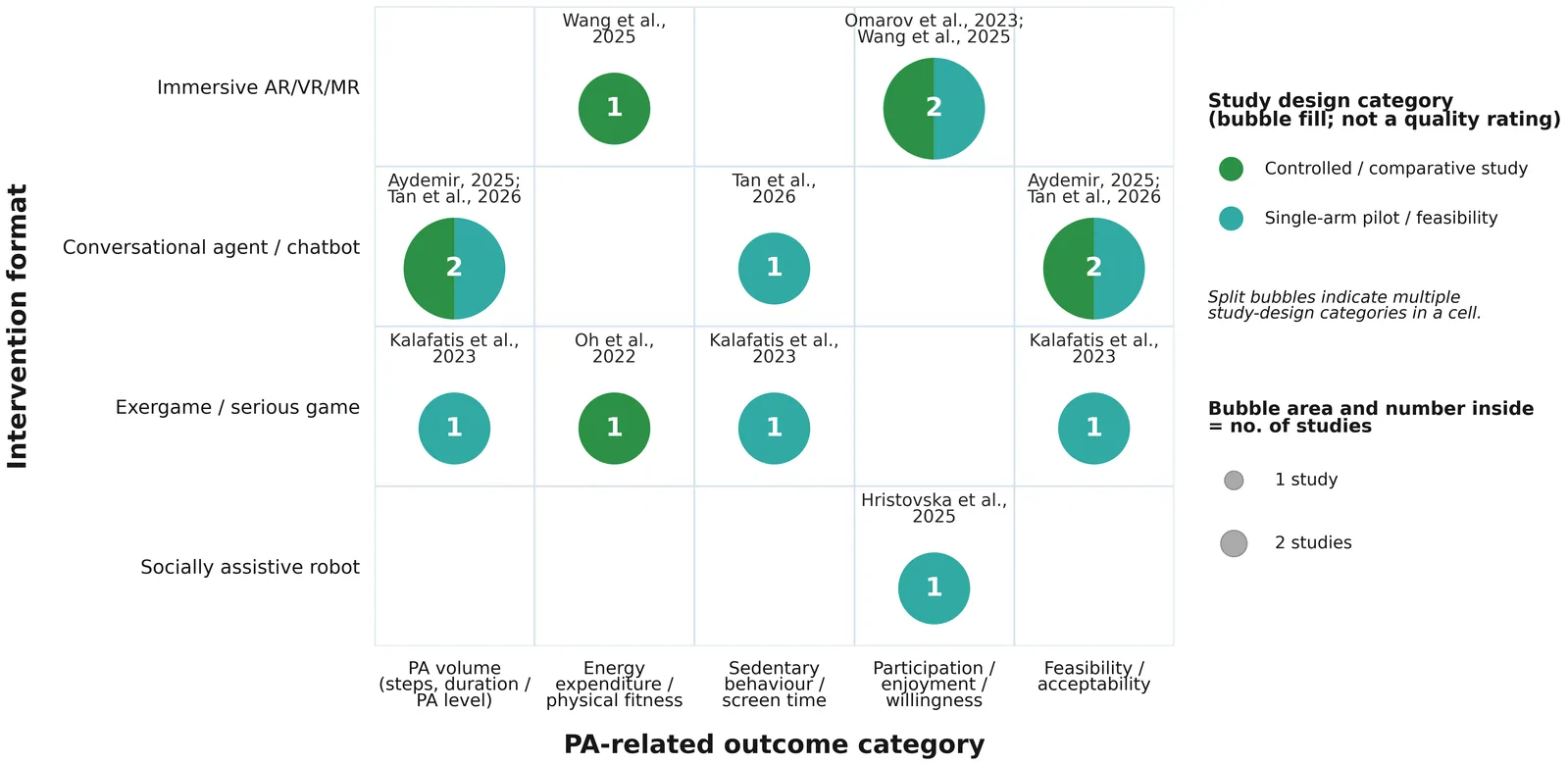
Evidence-gap map of AI-enabled physical activity interventions for children and adolescents (n = 7). Outcome categories are not mutually exclusives.

An evidence-gap map charted studies by intervention format and PA-related outcome category (Table 4; Figure 4). Outcome categories were non-mutually exclusive, so one study could appear in several cells. Bubble colour identifies the study-design category or categories represented within a cell; split colours indicate that more than one design category was present. The colours are descriptive and are not ratings of methodological quality or risk of bias.

PA volume was represented across conversational agents and serious games [27,29,32]. Energy expenditure or fitness appeared in an exergame and immersive VR [26,31], while sedentary behaviour or screen time appeared in one serious game and one chatbot study [27,32]. Participation, enjoyment, or willingness was evaluated in immersive systems and the robotic coach [28,30,31]. Controlled designs were present in only a limited subset of cells [26,29,31], and no included study directly isolated the added effect of an AI component against an otherwise matched non-AI version.

Taken together, the maps identify five principal gaps in this small and heterogeneous evidence base:

- Rigorous controlled evidence is scarce, with only one large randomized trial with follow-up [31] and two smaller controlled studies [26,29];
- Sedentary behaviour and screen-time outcomes were evaluated in only two studies [27,32];
- Objective device-based measurement of PA volume was limited, and several studies relied on self-report, feasibility, or experiential outcomes;
- Socially assistive robot formats lacked controlled evaluations [30]; and
- Most technique–format combinations remain untested, with generative AI confined to conversational formats [29,32] and reinforcement learning represented only in immersive VR [31].

## 4. Discussion

### 4.1. Principal Findings

This review identified seven reports published between 2022 and 2026 [26,27,28,29,30,31,32]. The evidence was small and heterogeneous: three studies used controlled designs and four were single-arm pilot or feasibility evaluations. AI served diverse technical roles, but PA and health outcomes were measured inconsistently, sedentary behaviour was rarely assessed, and only one large randomized trial included follow-up. The central finding is therefore not established effectiveness but an early-stage evidence base in which technical development has outpaced rigorous evaluation.

### 4.2. Interpretation and Comparison with Existing Reviews

The studies demonstrate several potential AI mechanisms: real-time movement recognition [26,28,30], periodic selection of individualized game content [27], generation of parent-mediated activity plans [29], adaptive virtual coaching [31], and context-aware student feedback [32]. These functions should be distinguished from outcomes. A system’s capacity to personalize content does not establish better adherence or engagement; perceived enjoyment or acceptability does not establish increased PA; and changes in PA, fitness, or body composition cannot be attributed specifically to AI unless the AI component is compared with an otherwise similar non-AI intervention. None of the included studies fully isolated that added contribution.

Earlier reviews were organized around wearable trackers [13], exergames [14], gamification [19], or immersive virtual reality [15], and generally treated AI as incidental. The present review complements those syntheses by mapping AI functions across formats and confirms their observation that rigorous evidence for technology-based PA interventions in young people remains limited and heterogeneous [13,14]. A related AI-mixed-reality prototype, Playground+ [33], illustrates a possible future direction for family PA play but was excluded from the evidence base because the extended abstract reported participant evaluation as future work and provided no eligible empirical PA-related outcome. This distinction between promising system concepts and evaluated interventions is important as the field develops.

### 4.3. Strengths and Limitations

This review followed PRISMA-ScR and JBI guidance, applied explicit eligibility criteria and standardized exclusion codes, and searched six databases spanning biomedical, engineering, and computing literature. The PCC framework, expanded AI-specific data charting, and technique–format and outcome-design maps enabled the functional role of AI and the distribution of evidence to be described systematically.

Several limitations should be considered. First, the small and heterogeneous evidence base precluded quantitative synthesis, and because no formal critical appraisal or risk-of-bias assessment was conducted, the study-design categories should not be interpreted as quality ratings. Second, the protocol was registered after database searching and screening, during data analysis, rather than prospectively. Third, although six major databases were searched using database-specific strategies, the focused AI terminology, restriction to English-language publications published from 2018 onward, and database-only study identification may have resulted in some relevant studies being missed. Given the rapid development of AI technologies, studies published after the final search date may also be absent. Finally, for mapping purposes, each hybrid intervention was assigned to a primary AI technique and intervention format, which may underrepresent the complexity of multicomponent systems. Incomplete reporting in the included studies also limited comparisons of model development, safety, privacy, fairness, and explainability.

### 4.4. Implications and Future Directions

Future evaluations should use adequately powered controlled designs, appropriate comparators, longer follow-up, and objective standardized PA and sedentary-behaviour measures. Comparisons with matched non-AI versions are needed to determine the added value of AI rather than the effect of the broader intervention package. Reports should specify model name and version, architecture, training and validation data, inputs, outputs, personalization mechanism, adaptation timing, performance, human oversight, error handling, safety constraints, data governance, fairness assessment, and explainability. Future research should consider co-design with young people, families, educators, and clinicians and should implement explicit safeguards for privacy, inappropriate output, movement safety, and equitable access [34,35].

For clinicians, educators, and policymakers, these technologies should currently be regarded as early-stage approaches to adaptive intervention delivery rather than established effective interventions. Implementation outside research settings should be accompanied by human oversight, safeguarding, and ongoing evaluation.

## 5. Conclusions

Seven studies mapped in this review used AI for movement recognition, content generation, feedback, or adaptive coaching in PA interventions for children and adolescents. The evidence was predominantly early-stage, heterogeneous, and insufficient to determine whether AI improves engagement, PA, sedentary behaviour, or health outcomes beyond comparable non-AI approaches. Rigorous controlled studies with objective outcomes and transparent AI-specific safety and reporting are required before effectiveness or readiness for widespread implementation can be established.

## Figures and Tables

**Table 1:** Full electronic search strategy for PubMed.

Concept Block	Search Terms/Limits
#1 Artificial intelligence	Artificial Intelligence OR Machine Learning OR Deep Learning OR Deep Neural Network OR Deep Reinforcement Learning OR AI Coach OR Computational Intelligence
#2 Physical activity/sedentary behaviour	Physical Activity OR Exercise OR Sedentary Behaviour OR Active Play OR Physical Activity Monitoring OR Screen Time OR Resistance Training OR Exercise Intervention OR Lifestyle Intervention OR Sport-based Intervention
#3 Children/adolescents	Child OR Children OR Adolescent OR Youth OR Teenager OR Pediatric OR School-age OR Childhood
Combination	#1 AND #2 AND #3
Limits	Publication date: 1 January 2018 to 24 May 2026; language: English
Records identified	1861

Note: Search executed on 24 May 2026. Terms within each concept block were combined with OR, and the three blocks were combined with AND. The complete database-specific strategies as executed are provided in Supplementary Tables S1–S6.

**Table 2:** Characteristics of the included studies (n = 7) on AI-enabled physical activity intervention strategies for children and adolescents.

Study (Author, Year) [Setting]	Study Design	Participants (n; Age; Population)	AI Approach	Intervention Format	PA-Related Outcome(s) [Measure]	Key Findings
Oh et al., 2022 [26] (Republic of Korea)	Two-group randomized comparative trial (3 weeks)	n = 24; adolescents aged 10–17 y who were overweight or obese	Deep learning (CNN-based gesture recognition)	AI motion-recognition home exergame (SUKIA), 30 min/session, 5 days/week; comparator: Nintendo Switch Ring Fit Adventure	Calorie expenditure; VO_2_max; 6MWT; BMI; RPE	Compared with Nintendo Switch, SUKIA produced higher calorie expenditure (*p* = 0.03), a larger VO_2_max improvement (time × group *p* = 0.005), and higher RPE (*p* = 0.04); both groups improved BMI and 6MWT.
Kalafatis et al., 2023 [27] (Greece)	Single-arm pre-pilot study (12 weeks)	n = 20; children aged 6–14 y with obesity	Evolutionary algorithm (genetic-algorithm procedural content generation)	ENDORSE serious game and parent app with individualized missions/messages plus a Fitbit tracker	Daily steps and sedentary time [Fitbit]; content acceptance/usefulness [parent questionnaire]	Mean daily steps were 9840.6. PA-message frequency was inversely correlated with steps (*p* = 0.042; combined PA/sedentary messages, *p* = 0.030), indicating adaptation to sensor data; pre-post PA effects were not tested.
Omarov et al., 2023 [28] (Kazakhstan)	Single-arm pilot study (one 30 min session)	n = 20; children aged 7–12 y	Computer-vision action recognition and machine-learning-based difficulty adjustment (TensorFlow)	AR motion-controlled Subway Surfers exergame (running, lateral movement, crouching, and jumping)	Self-reported enjoyment and willingness to engage in PA [instrument NR]	The authors reported greater enjoyment and willingness to engage in PA after gameplay; numerical estimates and comparator details were not reported.
Aydemir, 2025 [29] (Turkey)	Two-group randomized pre-post feasibility study (4 weeks)	n = 26 parent–child dyads; children with ASD (mean age approximately 14 y)	Generative AI/large language model (ChatGPT 4.0)	Parent-mediated, ChatGPT-generated home PA programme, 40 min/session, 3 days/week	PA level [LTEQ]; feasibility [parent questionnaire]	LTEQ scores increased from 6.69 to 34.00 in the intervention group versus 7.46 to 7.23 in controls (group × time *p* < 0.001); parents considered the activities feasible, interesting, and useful.
Hristovska et al., 2025 [30] (North Macedonia)	Single-session pilot evaluation	n = 10; healthy children aged 9–11 y	Deep-learning pose estimation and keypoint detection (MediaPipe)	Alpha 1S humanoid robotic coach with five guided exercises and real-time movement feedback	PA enjoyment [4-item short PACES]	Children reported high exercise enjoyment (item means 4.4–4.8/5); PA levels or longer-term effects were not assessed.
Wang et al., 2025 [31] (China)	Five-arm RCT (8 weeks; 6-month follow-up)	n = 227 analyzed; adolescents aged 11–17 y with excess body weight (mean 14.24 y)	Deep reinforcement learning-trained transformer virtual coaching agent	Immersive VR table tennis or soccer with an AI coach, 45 min/session, 3 days/week (REVERIE); physical-sports and usual-PE comparators	Fat mass (primary); physical fitness; sports willingness; psychological well-being	REVERIE reduced fat mass by 4.28 kg versus control and did not differ significantly from physical sports; fitness, sports willingness, and well-being improved, with benefits maintained at 6 months in the REVERIE group.
Tan et al., 2026 [32] (USA)	Single-arm pilot feasibility study (8 weeks)	n = 172; middle-school students (mostly Grade 6; approximately 12 y)	GPT-4-generated context-aware feedback combined with a rule-based goal-attainment engine	School-based ProudMe Tech web app for goal setting, self-monitoring, reflection, and personalized feedback	PA and screen time [self-report]; engagement and goal attainment	The app was feasible and engaged students; screen time decreased by 21.6%; whereas, PA did not change significantly.

Note: Studies are listed chronologically; bracketed locations denote the study setting where reported. Abbreviations: 6MWT, 6 min walk test; AI, artificial intelligence; AR, augmented reality; ASD, autism spectrum disorder; BMI, body mass index; CNN, convolutional neural network; LTEQ, Leisure-Time Exercise Questionnaire; NR, not reported; PA, physical activity; PACES, Physical Activity Enjoyment Scale; RCT, randomized controlled trial; RPE, rating of perceived exertion; VO_2_max, maximal oxygen uptake; VR, virtual reality.

**Table 3:** Cross-tabulation of AI technique family by intervention format among the seven included studies.

AI Technique Family	Immersive AR/VR/MR	Conversational Agent/Chatbot	Exergame/Serious Game	Socially Assistive Robot	Total
Computer vision/pose and motion recognition	[28] (1)	-	[26] (1)	[30] (1)	3
Generative AI/large language models	-	[29,32] (2)	-	-	2
Reinforcement learning (+ language agent)	[31] (1)	-	-	-	1
Evolutionary computation	-	-	[27] (1)	-	1
Total	2	2	2	1	7

Note: Cell entries are reference-list citation numbers; figures in parentheses are study counts. Each study was assigned one primary AI technique and one primary intervention format; hybrid features are described in Section 3.3 and Section 3.4 and Supplementary Table S9. Hyphens indicate unrepresented combinations. References: [26] Oh et al., 2022; [27] Kalafatis et al., 2023; [28] Omarov et al., 2023; [29] Aydemir, 2025; [30] Hristovska et al., 2025; [31] Wang et al., 2025; [32] Tan et al., 2026.

**Table 4:** Evidence-gap map: intervention format by category of PA-related outcome among the seven included studies.

Intervention Format	PA Volume (Steps, Duration, PA Level)	Energy Expenditure/Physical Fitness	Sedentary Behaviour/Screen Time	Participation/Enjoyment/Willingness	Feasibility/Acceptability
Immersive AR/VR/MR	-	[31]	-	[28,31]	-
Conversational agent/chatbot	[29,32]	-	[32]	-	[29,32]
Exergame/serious game	[27]	[26]	[27]	-	[27]
Socially assistive robot	-	-	-	[30]	-

Note: Cell entries are reference-list citation numbers. Outcome categories are not mutually exclusive, so a study may appear in more than one cell. Hyphens indicate unrepresented intervention-outcome combinations. Study-design categories shown in Figure 4 are descriptive and are not ratings of methodological quality or risk of bias. References are listed in the note to Table 3.

## Data Availability

No new data were created or analyzed in this study. Data sharing is not applicable to this article. The registered review protocol is available at https://doi.org/10.37766/inplasy2026.6.0095 (INPLASY202660095).

## Supplementary Materials

The following supporting information can be downloaded at: https://www.mdpi.com/article/10.3390/children13081114/s1, Tables S1–S6: Complete database-specific search strategies as executed; Table S7: PRISMA-ScR checklist; Table S8: Expanded data-charting form; Table S9: Functional role and reported technical characteristics of AI in the seven included studies.

## Abbreviations

The following abbreviations are used in this manuscript:

AI	Artificial intelligence
PA	Physical activity
MVPA	Moderate-to-vigorous physical activity
PRISMA-ScR	Preferred Reporting Items for Systematic Reviews and Meta-Analyses extension for Scoping Reviews
JBI	Joanna Briggs Institute
PCC	Population-Concept-Context
INPLASY	International Platform of Registered Systematic Review and Meta-Analysis Protocols
RCT	Randomized controlled trial
AR	Augmented reality
VR	Virtual reality
MR	Mixed reality
LLM	Large language model
CNN	Convolutional neural network
ASD	Autism spectrum disorder
BMI	Body mass index
VO_2_max	Maximal oxygen uptake
6MWT	Six-minute walk test
RPE	Rating of perceived exertion
