# Supplementary material for: AI-Enabled Physical Activity Intervention Strategies for Children and Adolescents: A Scoping Review
Source: Children (Basel). 2026 Aug 20;13(8):1114. doi: 10.3390/children13081114 (PMC13510757; doi:10.3390/children13081114)
Supplement: Supplementary file 1 [file children-13-01114-s001.zip › children-4419301-supplementary.pdf]

Supplementary Materials

S1. Database Search Strategies

Six electronic databases were searched on 24 May 2026. The executed search combined three concept blocks (artificial intelligence; physical activity or sedentary behaviour; children or adolescents) with AND, with synonyms within each block combined with OR. Date (1 January 2018 onward) and language (English) limits were applied. Tables S1-S6 reproduce the database-specific queries and record counts used in the review.

Table S1. Search strategy for PubMed.

**Database**	PubMed
**Date searched**	24 May 2026
**Field/limits**	Date: 1 January 2018 to 24 May 2026; language: English
**Search query**	(Artificial Intelligence OR Machine Learning OR Deep Learning OR Deep Neural Network OR Deep Reinforcement Learning OR AI Coach OR Computational Intelligence) AND (Physical Activity OR Exercise OR Sedentary Behavior OR Active Play OR Physical Activity Monitoring OR Screen Time OR Resistance Training OR Exercise Intervention OR Lifestyle Intervention OR Sport-based Intervention) AND (Child OR Children OR Adolescent OR Youth OR Teenager OR Pediatric OR School-age OR Childhood)
**Records identified (before limits)**	2200
**Records after date and language limits**	1861
**Export format**	RIS

Table S2. Search strategy for Web of Science Core Collection.

**Database**	Web of Science Core Collection
**Date searched**	24 May 2026
**Field/limits**	Date: 1 January 2018 to 24 May 2026; language: English
**Search query**	(Artificial Intelligence OR Machine Learning OR Deep Learning OR Deep Neural Network OR Deep Reinforcement Learning OR AI Coach OR Computational Intelligence) AND (Physical Activity OR Exercise OR Sedentary Behavior OR Active Play OR Physical Activity Monitoring OR Screen Time OR Resistance Training OR Exercise Intervention OR Lifestyle Intervention OR Sport-based Intervention) AND (Child OR Children OR Adolescent OR Youth OR Teenager OR Pediatric OR School-age OR Childhood) [Topic search]
**Records identified (before limits)**	2339
**Records after date and language limits**	2213
**Export format**	RIS

Table S3. Search strategy for Scopus.

**Database**	Scopus
**Date searched**	24 May 2026
**Field/limits**	Date: 1 January 2018 to 24 May 2026; language: English
**Search query**	("Artificial Intelligence" OR "Machine Learning" OR "Deep Learning" OR "Deep Neural Network" OR "Deep Reinforcement Learning" OR "AI Coach" OR "Computational Intelligence") AND ("Physical Activity" OR "Exercise" OR "Sedentary Behavior" OR "Active Play" OR "Physical Activity Monitoring" OR "Screen Time" OR "Resistance Training" OR "Exercise Intervention" OR "Lifestyle Intervention" OR "Sport-based Intervention") AND (child* OR adolescent* OR youth* OR teenager* OR pediatric* OR "School-age" OR childhood) [TITLE-ABS-KEY]
**Records identified (before limits)**	1293
**Records after date and language limits**	1187
**Export format**	RIS

Table S4. Search strategy for Embase.

**Database**	Embase
**Date searched**	24 May 2026
**Field/limits**	Date: 1 January 2018 to 24 May 2026; language: English
**Search query**	('artificial intelligence'/exp OR 'machine learning'/exp OR 'deep learning'/exp OR 'neural network'/exp OR 'reinforcement learning'/exp OR 'computational intelligence'/exp OR 'artificial intelligence':ti,ab,kw OR 'machine learning':ti,ab,kw OR 'deep learning':ti,ab,kw OR 'deep neural network*':ti,ab,kw OR 'deep reinforcement learning':ti,ab,kw OR 'ai coach*':ti,ab,kw OR 'computational intelligence':ti,ab,kw) AND ('physical activity'/exp OR 'exercise'/exp OR 'sedentary lifestyle'/exp OR 'sedentary behavior'/exp OR 'active play'/exp OR 'screen time'/exp OR 'resistance training'/exp OR 'lifestyle intervention'/exp OR 'physical activity':ti,ab,kw OR 'exercise*':ti,ab,kw OR 'sedentary behavio*':ti,ab,kw OR 'active play':ti,ab,kw OR 'physical activity monitoring':ti,ab,kw OR 'screen time':ti,ab,kw OR 'resistance training':ti,ab,kw OR 'exercise intervention*':ti,ab,kw OR 'lifestyle intervention*':ti,ab,kw OR 'sport* based intervention*':ti,ab,kw) AND ('child'/exp OR 'adolescent'/exp OR 'youth'/exp OR 'pediatrics'/exp OR 'school child'/exp OR 'child*':ti,ab,kw OR 'adolescent*':ti,ab,kw OR 'youth*':ti,ab,kw OR 'teenager*':ti,ab,kw OR 'pediatric*':ti,ab,kw OR 'school age*':ti,ab,kw OR 'childhood':ti,ab,kw)
**Records identified (before limits)**	2733
**Records after date and language limits**	2079
**Export format**	RIS

Table S5. Search strategy for IEEE Xplore.

**Database**	IEEE Xplore
**Date searched**	24 May 2026
**Field/limits**	Date: 1 January 2018 to 24 May 2026; language: English
**Search query**	(Artificial Intelligence OR Machine Learning OR Deep Learning OR Deep Neural Network OR Deep Reinforcement Learning OR AI Coach OR Computational Intelligence) AND (Physical Activity OR Exercise OR Sedentary Behavior OR Active Play OR Physical Activity Monitoring OR Screen Time OR Resistance Training OR Exercise Intervention OR Lifestyle Intervention OR Sport-based Intervention) AND (Child OR Children OR Adolescent OR Youth OR Teenager OR Pediatric OR School-age OR Childhood) [All metadata]
**Records identified (before limits)**	957
**Records after date and language limits**	837
**Export format**	CSV

Table S6. Search strategy for ACM Digital Library.

**Database**	ACM Digital Library
**Date searched**	24 May 2026
**Field/limits**	Date: 1 January 2018 to 24 May 2026; language: English
**Search query**	[[Title: "artificial intelligence"] OR [Title: "machine learning"] OR [Title: "deep learning"] OR [Title: "deep neural network"] OR [Title: "deep reinforcement learning"] OR [Title: "ai coach"] OR [Title: "computational intelligence"] OR [Abstract: "artificial intelligence"] OR [Abstract: "machine learning"] OR [Abstract: "deep learning"] OR [Abstract: "deep neural network"] OR [Abstract: "deep reinforcement learning"] OR [Abstract: "ai coach"] OR [Abstract: "computational intelligence"]] AND [[Title: "physical activity"] OR [Title: exercise] OR [Title: "sedentary behavior"] OR [Title: "active play"] OR [Title: "physical activity monitoring"] OR [Title: "screen time"] OR [Title: "resistance training"] OR [Title: "exercise intervention"] OR [Title: "lifestyle intervention"] OR [Title: "sport-based intervention"] OR [Abstract: "physical activity"] OR [Abstract: exercise] OR [Abstract: "sedentary behavior"] OR [Abstract: "active play"] OR [Abstract: "physical activity monitoring"] OR [Abstract: "screen time"] OR [Abstract: "resistance training"] OR [Abstract: "exercise intervention"] OR [Abstract: "lifestyle intervention"] OR [Abstract: "sport-based intervention"]] AND [[Title: child] OR [Title: children] OR [Title: adolescent] OR [Title: youth] OR [Title: teenager] OR [Title: pediatric] OR [Title: "school-age"] OR [Title: childhood] OR [Abstract: child] OR [Abstract: children] OR [Abstract: adolescent] OR [Abstract: youth] OR [Abstract: teenager] OR [Abstract: pediatric] OR [Abstract: "school-age"] OR [Abstract: childhood]] AND [E-Publication Date: (01/01/2018 TO 05/24/2026)]
**Records identified (before limits)**	1110
**Records after date and language limits**	792
**Export format**	RIS/BibTeX

Across the six databases, 10,632 records were identified before limits and 8,969 after the date and language limits (PubMed, 1,861; Web of Science Core Collection, 2,213; Scopus, 1,187; Embase, 2,079; IEEE Xplore, 837; ACM Digital Library, 792). After removal of 1,990 duplicate records, 6,979 records proceeded to title and abstract screening. Forty-eight reports were sought for retrieval; one was not retrieved, and 47 were assessed for eligibility. Forty reports were excluded (population not eligible, n = 6; no AI component, n = 8; no eligible PA-related outcome, n = 14; ineligible publication type, n = 12), leaving 7 studies in the review.

S2. PRISMA-ScR Checklist

Table S7. PRISMA-ScR checklist for the scoping review.

#	Section / item	Checklist item (brief)	Reported in (section)
1	Title	Identified as a scoping review.	Title
2	Abstract	Structured summary: background, objectives, eligibility, sources, charting methods, results, conclusions.	Abstract
3	Rationale	Rationale for the review in the context of current knowledge.	Section 1
4	Objectives	Review questions/objectives, with the PCC framework.	Section 1
5	Protocol and registration	Protocol existence and registration details.	Section 2.1
6	Eligibility criteria	Source characteristics used as eligibility criteria.	Section 2.2
7	Information sources	All information sources and last search date.	Section 2.3
8	Search	Full electronic search strategy for at least one database.	Section 2.4; Table 1; Tables S1-S6
9	Selection of sources	Process for selecting sources (screening, eligibility).	Section 2.5; Figure 1
10	Data charting process	Method and process of data charting.	Section 2.6; Table S8
11	Data items	Variables for which data were charted, and definitions.	Section 2.7; Table S8
12	Critical appraisal (optional)	Methods for appraising sources, if done.	Section 2.8 (not performed)
13	Synthesis of results	Methods of handling and summarizing charted data.	Section 2.9
14	Selection of sources	Numbers screened, assessed, and included, with exclusions.	Section 3.1; Figure 1
15	Characteristics of sources	Characteristics of the charted sources.	Section 3.2; Table 2
16	Critical appraisal (optional)	Appraisal results, if conducted.	Not applicable (Section 2.8)
17	Results of individual sources	Charted data relevant to the review questions.	Sections 3.2-3.8; Tables 2-4
18	Synthesis of results	Summary/organization of charted results vs objectives.	Sections 3.3-3.8; Figures 2-4
19	Summary of evidence	Summary of main results, linked to objectives and audiences.	Section 4.1
20	Limitations	Limitations of the scoping review process.	Section 4.3
21	Conclusions	General interpretation, implications, and next steps.	Sections 4.4 and 5
22	Funding	Sources of funding and role of funders.	Funding statement

Note: Checklist items are summarized from Tricco et al. (PRISMA-ScR; Ann. Intern. Med. 2018, 169, 467-473). Items 12 and 16 (critical appraisal) are optional and were not undertaken, consistent with the mapping aim of this review.

S3. Data-Charting Framework

Table S8. Expanded data-charting form: variables charted for each included study.

Data item	Operational description
First author, year	First author surname and year of publication.
Country / setting	Country and home, school, clinical, community, laboratory, or mixed setting.
Study design and comparator	Design, number of groups, allocation method, comparator, follow-up, and evidence type.
Participant characteristics	Sample size, age or age range, sex where reported, and target population.
Intervention dose	Duration, session frequency, and session length.
AI approach / technique	Computer vision, machine learning, generative AI/LLM, reinforcement learning, evolutionary computation, or hybrid approach.
Model name and version	Named model, platform, framework, and version, where reported.
Model architecture	Reported architecture or algorithmic method, including custom or pretrained status.
Training and validation data	Source, sample, labels, and validation data used to develop or tune the AI component.
Input data / sensors	Prompts, self-reports, activity trackers, cameras, pose landmarks, VR states, game interactions, or other inputs.
AI output / decision	Classification, feedback, recommendation, generated content, difficulty adjustment, coaching action, or other output.
Functional role of AI	How AI affected delivery, personalization, feedback, monitoring, decision-making, or analysis.
Personalization mechanism	Rules or learned mechanism linking individual data to tailored content or feedback.
Timing of adaptation	Pre-intervention, offline/batch, periodic, or real-time adaptation.
Model performance	Accuracy, reliability, latency, usability, or other technical performance metrics.
AI-specific comparator	Comparison with a non-AI version, alternative algorithm, ablation, or conventional intervention.
Human oversight	Role of researchers, parents, teachers, clinicians, coaches, or other human reviewers.
Error handling and safety	Moderation, unsafe-output handling, movement-safety controls, injury or cybersickness monitoring, and escalation procedures.
Data storage and privacy	Data flow, deidentification, access control, encryption, storage, and platform/API handling.
Bias / fairness assessment	Assessment across demographic or functional subgroups, where reported.
Explainability / transparency	User-facing explanations, rule visibility, or interpretability measures.
Intervention format / delivery	Immersive AR/VR/MR, conversational agent/chatbot, exergame/serious game, socially assistive robot, mobile/web app, or hybrid delivery.
Participant exposure to AI	Whether participants interacted directly with AI or received AI-generated content through a human.
PA-related outcome(s)	PA volume, energy expenditure/fitness, sedentary/screen time, participation/enjoyment/willingness, and feasibility/acceptability.
Measurement tool(s)	Objective sensors, validated questionnaires, self-report, anthropometry, fitness tests, or system logs.
Principal findings	Main PA-related, feasibility, engagement, and technical results, with uncertainty or numerical estimates where reported.
Limitations relevant to interpretation	Design, sample, duration, comparator, measurement, and AI-reporting limitations.

Note: This table presents the data-charting framework used in the review; not every item was applicable to or reported in every included study. Core study and outcome variables were specified before final charting, and the form was refined iteratively to capture AI-specific technical and governance characteristics. Items not reported by a source were recorded as NR.

Table S9. Functional role and reported technical characteristics of AI in the 7 included studies.

Study	AI component / model	Input data	AI output / decision	Functional role and timing	Technical evaluation / comparator	Human oversight, safety, and data governance
Oh et al., 2022 [26]	CNN-based gesture-recognition module in SUKIA; architecture, training data, and version NR	Real-time smartphone-camera images of player gestures	Gesture classification drove the game character, scoring, and audiovisual feedback	Direct, real-time AI-enabled exercise delivery	Randomized comparison with Nintendo Switch Ring Fit Adventure; no AI ablation and no recognition-accuracy metric reported	IRB approval and supervised study procedures reported; AI-specific safety, error handling, and data governance NR
Kalafatis et al., 2023 [27]	Genetic-algorithm procedural content generation in ENDORSE	Fitbit steps, sedentary time, and sleep; game/app interaction data	Selected weekly individualized missions and educational/progress messages	Periodic weekly adaptation across 12 iterations	Parent acceptance/usefulness ratings and correlations between sensor status and message frequency; no non-AI comparator	Healthcare professionals designed the messages; AI-specific error handling, fairness, and privacy safeguards NR
Omarov et al., 2023 [28]	TensorFlow-based computer vision/action recognition with machine-learning difficulty adjustment; specific model NR	Real-time camera video and 33 body landmarks / pose keypoints	Movement commands (left, right, jump, crouch, run) and adaptive gameplay difficulty	Direct, real-time motion-controlled AR gameplay	Pose demonstrations and a brief user pilot with self-reported enjoyment/willingness; no numerical recognition accuracy or non-AI comparator	Safety and recognition accuracy were identified as future challenges; implemented safeguards, privacy, and data storage procedures NR
Aydemir, 2025 [29]	ChatGPT 4.0	Standardized parent prompts incorporating the child's age, ASD-related needs, and home context	Text-based, age-appropriate home PA plans and implementation guidance	Content was generated before and during the 4-week intervention; parents delivered the activities and could reprompt ChatGPT	Randomized intervention-versus-control feasibility study; no AI-specific performance test or non-AI content-generation comparator	Parents received ChatGPT training; researcher answered questions through WhatsApp; prompts included home-safety considerations; study data were anonymized, but platform-level AI data handling was NR
Hristovska et al., 2025 [30]	Pretrained MediaPipe pose-estimation / keypoint-detection models	Real-time computer-camera video of five predefined exercises	Pose correctness decision, correct-repetition count, and visual feedback	Direct, real-time movement monitoring alongside robotic demonstration and vocal motivation	Single-session user evaluation of enjoyment; no model-accuracy metric, non-AI comparator, or longer-term evaluation	Exercises and robot demonstrations were predefined by the developers; AI-specific error handling, privacy, and data storage NR
Wang et al., 2025 [31]	Transformer-based REVERIE Coach trained with two-stage template-driven, feedback-oriented deep reinforcement learning; RTMDet-Ins used in rendering	VR positions, rotations, impact forces, ball states, coach templates, and adolescent-AI interaction data	Adaptive sports actions, empathetic technique guidance, personalized instructions, and coach-schedule support	Predeployment digital-twin training followed by optimization using AI-human interaction data during deployment	Comparative system testing, pilot user testing, and a five-arm RCT versus physical sports and usual PE; biomechanical comparability, exercise intensity, and heart rate evaluated	Professional-coach panel created and refined templates; human-assistance module supported schedules; cybersickness and injuries monitored; privacy and fairness assessment NR

Study	AI component / model	Input data	AI output / decision	Functional role and timing	Technical evaluation / comparator	Human oversight, safety, and data governance
Tan et al., 2026 [32]	OpenAI GPT-4 API combined with a rule-based goal-attainment engine; temperature 0.9, max_tokens 75	Student-set goals, self-reported behaviours, reflections, and goal-attainment percentages	Real-time, context-aware feedback: praise, educational prompts, or constructive suggestions	Direct real-time feedback after user entries; tone was conditioned on goal achievement	System-log feasibility study; 5,804 feedback messages analyzed; no non-AI chatbot comparator	System prompts constrained tone; OpenAI Moderation API filtered content; throttling and an environment-stored API key were used; study data were deidentified and stored on encrypted, password-protected servers

Note: AI characteristics are reported only when stated in the source article. NR, not reported; AI, artificial intelligence; AR, augmented reality; CNN, convolutional neural network; PE, physical education; RCT, randomized controlled trial.
